# Soluble B7-CD28 Family Inhibitory Immune Checkpoint Proteins and Anti-Cancer Immunotherapy

**DOI:** 10.3389/fimmu.2021.651634

**Published:** 2021-08-31

**Authors:** Muhammad Khan, Sumbal Arooj, Hua Wang

**Affiliations:** ^1^Department of Oncology, the First Affiliated Hospital of Anhui Medical University, Hefei, China; ^2^Inflammation and Immune Mediated Diseases Laboratory of Anhui Province, Anhui Medical University, Hefei, China; ^3^Department of Biochemistry, University of Sialkot, Sialkot, Pakistan

**Keywords:** soluble immune checkpoints (SIC), alternative splice variants (ASV), immunotherapy (IT), immune checkpoint blockade (ICB), gene therapy, cancer vaccination (CVax)

## Abstract

Co-inhibitory B7-CD28 family member proteins negatively regulate T cell responses and are extensively involved in tumor immune evasion. Blockade of classical CTLA-4 (cytotoxic T lymphocyte-associated antigen-4) and PD-1 (programmed cell death protein-1) checkpoint pathways have become the cornerstone of anti-cancer immunotherapy. New inhibitory checkpoint proteins such as B7-H3, B7-H4, and BTLA (B and T lymphocyte attenuator) are being discovered and investigated for their potential in anti-cancer immunotherapy. In addition, soluble forms of these molecules also exist in sera of healthy individuals and elevated levels are found in chronic infections, autoimmune diseases, and cancers. Soluble forms are generated by proteolytic shedding or alternative splicing. Elevated circulating levels of these inhibitory soluble checkpoint molecules in cancer have been correlated with advance stage, metastatic status, and prognosis which underscore their broader involvement in immune regulation. In addition to their potential as biomarker, understanding their mechanism of production, biological activity, and pathological interactions may also pave the way for their clinical use as a therapeutic target. Here we review these aspects of soluble checkpoint molecules and elucidate on their potential for anti-cancer immunotherapy.

## Introduction

**Figure 1:**
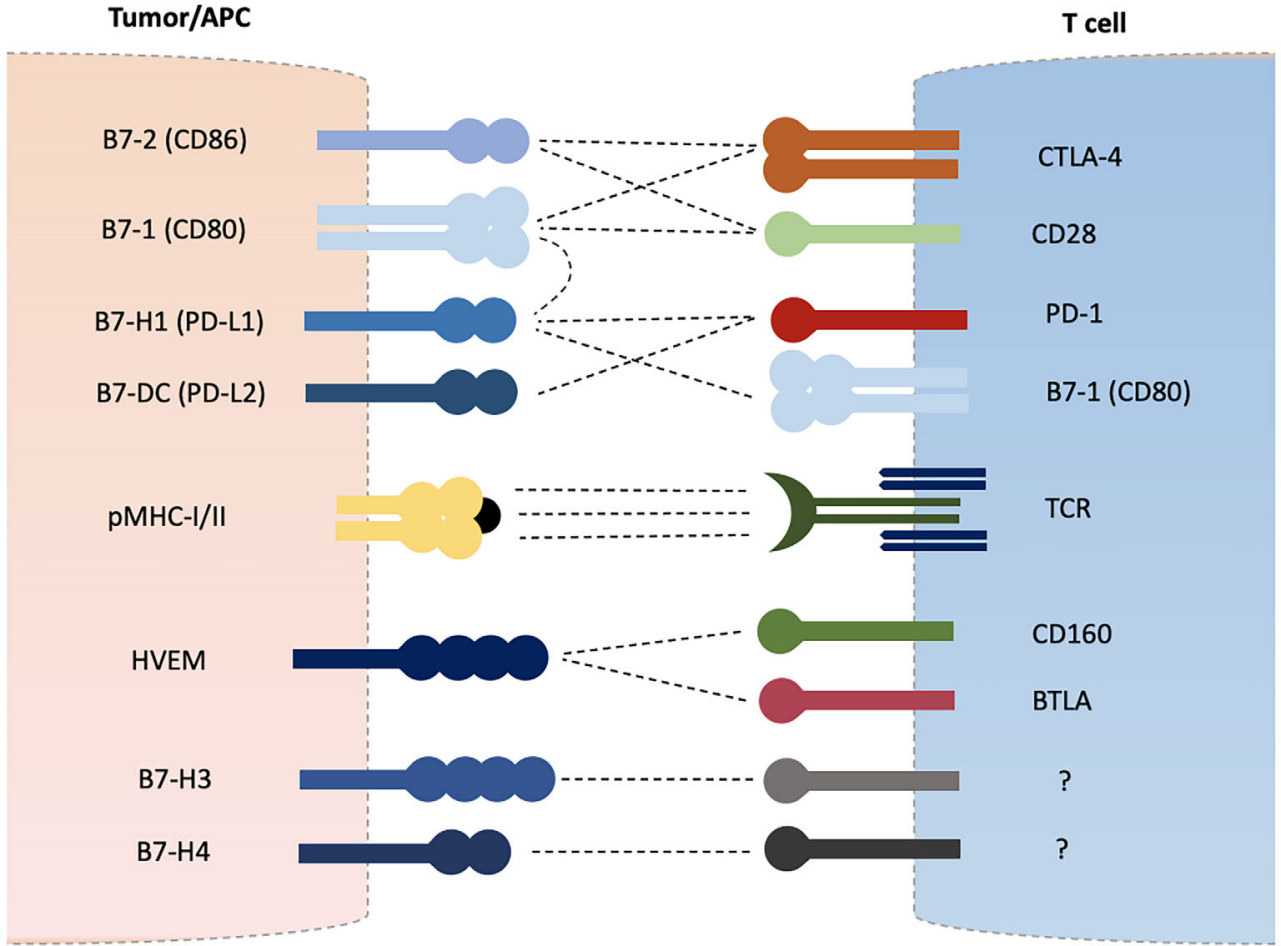
B7-CD28 Family Coinhibitory Checkpoint Molecules.

Adaptive immune system is equipped with T and B lymphocytes that are essential in maintaining self-tolerance and eliminating or destroying foreign harmful invaders (1). Antigens are presented to T cells by major histocompatibility complex class I or II molecules (MHC-I/II) expressed on normal cells or antigen presenting cells (APCs) resulting in T cell activation *via* peptide-MHC and T cell receptor (TCR) interaction (2, 3). A secondary signal is further required to induce T cell activation which is provided by costimulatory molecules such as CD28 and inducible T-cell co-stimulator (ICOS) which are termed as positive regulators of T cell functions (2–6). A third and final signal is provided in the form of various cytokines to direct and amplify T cell differentiation and expansion. Negative regulators such as cytotoxic T lymphocyte-associated antigen-4 (CTLA-4), programmed cell death protein-1 (PD-1), and B and T lymphocyte attenuator (BTLA) are upregulated after T cell activation in order to avoid overactivation and hyperactivity (6–11). These receptors constitute the CD28 receptor family which mainly recognizes B7 family proteins expressed on variety of cells including tumor cells and APCs as their ligands (12–14). CD28 recognize B7-1 (CD80) and B7-2 (CD86) as its ligands. CTLA-4 competes for the same ligands and cause T cell inhibition (4). The CTLA-4/CD28/B7-1/B7-2 group mainly affects the early phase of T cell activation (15). Similarly, PD-1 expressed on T cells recognizes B7-H1 (PD-L1) and B7-DC (PD-L2) as its ligands and results in inhibition of T cell effector functions and induces T cell apoptotic death (7–10). The PD-1/PD-L1/PD-L2 regulate the effector phase of T cell activation (15). Cancer cells manipulate these coinhibitory receptors in order to avoid destruction by immune system and blockade of such interactions through monoclonal antibodies have become the cornerstone of anti-cancer immunotherapy (16–21). Other newer costimulatory and coinhibitory molecules belonging to CD28-B7 family receptors are being discovered and investigated for their role in cancer immune evasion such as BTLA, B7-H3, B7-H4, and B7-H5, etcetera (11, 14, 22) (**Figure 1**). Of these, BTLA (also known as CD272) has shown some similarities with CTLA-4 and PD-1 in their regulatory effects on T cell activation and is the subject of intense investigations in recent times (11, 22–37). BTLA recognizes HVEM (herpes virus entry mediator, TNFRSF14, CD270) as its ligand and their interactions have shown to inhibit T cell activation and proliferation (22–28). BTLA is expressed on naïve as well as activated T cells which suggests it may regulate all phases of T cell activation as opposed to CTLA-4 (early naïve phase of T cell activation) and PD-1 (late effector phase) (22, 27, 28). Several cancers have shown up-regulation of BTLA and its blockade has displayed an enhanced immune response (29–37). Other newly discovered B7 ligands such as B7-H3, B7-H4 and B7-H5 have also shown to play inhibitory roles in T cell activation, and have demonstrated up-regulation in various cancers (12–14, 38, 39).

**Figure 2:**
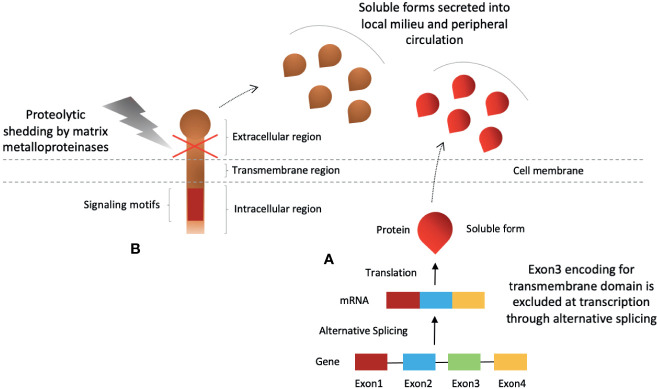
Production of soluble forms of CD28-B7 family coinhibitory immune checkpoint molecules. Soluble checkpoint proteins are produced by two mechanisms; **(A)** alternative splicing, and **(B)** proteolytic shedding of extracellular region.

Soluble forms of these molecules can be detected in plasma of healthy individuals that are either produced by shedding of the membrane form or through alternative splicing (29–32, 40–44) (**Figure 2**). Elevated plasma levels are reported in disease progression, autoimmune diseases and cancers (29–32, 39, 42). In recent times, investigation into the soluble forms of these molecules have been exaggerated. Although, the bulk of the reports are aimed at assessing their predictive and prognostic value, studies have also reported that they are biologically active and could hold potential for anti-cancer therapy (29–32, 40–45). We will review these soluble inhibitory checkpoints in detail with a focus on their potential for anti-cancer immunotherapy.

## CTLA-4 Inhibitory Checkpoint Molecules Axis

Cytotoxic T lymphocyte-associated antigen- 4 (CTLA-4), also known as CD152, is a type 1 transmembrane glycoprotein of the immunoglobin superfamily and member of CD28 family receptors (46). CTLA-4 molecule is comprised of 223 amino acids in length, with a 35 aa signal peptide, and found as a covalent homodimer of 41–43 kDa (46–49). CTLA-4 is expressed upon T cell activation following TCR engagement (48–50). In addition to activated and memory T cells, several other immune cells also express CTLA-4 such as regulatory T cells (Tregs; which constitutively express CTLA-4) and tumor-infiltrating NK cells, and is induced on mouse NK cells upon IL-2 stimulation (50–52). CTLA-4 competes with CD28 costimulatory molecule for binding to the same ligands – B7-1 (CD80) and B7-2 (CD86) (12, 53–56). Its ligation results in inhibition of T cells, production of IL-2, proliferation and survival (55–58). CTLA-4 has been well-established as a negative regulator of peripheral T cell tolerance and autoreactivity, and is involved cancer immune evasion (12, 55–59). Successful blockade of CTLA-4 with monoclonal antibody such as ipilimumab has shown improved outcome for cancer patients (60, 61). However, recently additional mechanisms have been proposed to explain the immunotherapeutic effect of anti-CTLA-4 mAbs including Fc receptor-dependent depletion of regulatory T (Treg) cells in tumor microenvironment, and blocking of trans-endocytosis of B7 on dendritic cells (DC) (62–66). Nonetheless, more studies are needed to confirm these findings. Recent studies have demonstrated increased levels of soluble counterparts of CTLA-4, B7-1, and B7-2 in the plasma of cancer patients (30, 31). Moreover, fold changes in the serum levels of these molecules revealed a positive correlation after treatment induction suggesting a regulatory interplay among the soluble forms in cohesion with membrane-bound counterparts (29, 30). Therefore, it seems that the CTLA-4/B7-1/B7-2 checkpoint pathway proteins in soluble forms may also play critical role in T cell regulation.

### Soluble CTLA-4

CTLA-4 gene, located on chromosome 2 in humans, consists of 4 exons that constitute the full length CTLA-4 molecule (flCTLA-4) (47, 67). Alternative splicing results in deletion of certain exons giving rise to four different splice variants; flCTLA-4, soluble CTLA-4 (lacking exon 3), transcripts coding for exons 1 and 4, and ligand-independent CTLA-4 (liCTLA-4 isoform lacking exon 2, thus unable to bind to its receptor, and is only found in mice) (40, 47, 67–70). Only one splice variant that lacks exon 3, which encodes for the transmembrane domain, is translated into soluble CTLA-4 form (sCTLA-4) (5, 40, 68). Unlike full length that is homodimer molecule, soluble CTLA-4 is produced as monomer (5, 40, 68). Soluble CTLA-4 can be detected in serum of healthy individuals (40, 68). In addition, hematolymphoid organs such as lymph nodes, spleen and blood, both in humans and rats, have shown sCTLA-4 expression (40). Peripheral blood lymphocytes such as regulatory T cells, non-activated T cells (both CD4+ and CD8+ T cells) as well as B lymphocytes have been reported to express the CTLA-4delTM (splice variant of CTLA-4) (5, 40, 68). Tregs were identified as the prominent source of sCTLA-4 (71). *In vitro* analysis of human T cells has shown that sCTLA-4 secretion can be increased during responses contradicting the previous reports of sCTLA-4 secretion in resting T cells (40, 68, 71–74). Melanoma cancer cell lines were also reported to produce sCTLA-4 (75). Genotype CT60 (A/G) was correlated with soluble CTLA-4 production as homozygous AA individuals express higher levels of mRNA at basal conditions as well as upon T cell stimulation (72). Thus, the expression profile of soluble CTLA-4 reflects its apparent role in adaptive immune responses and involvement in immune evasion of cancer cells.

**Figure 3:**
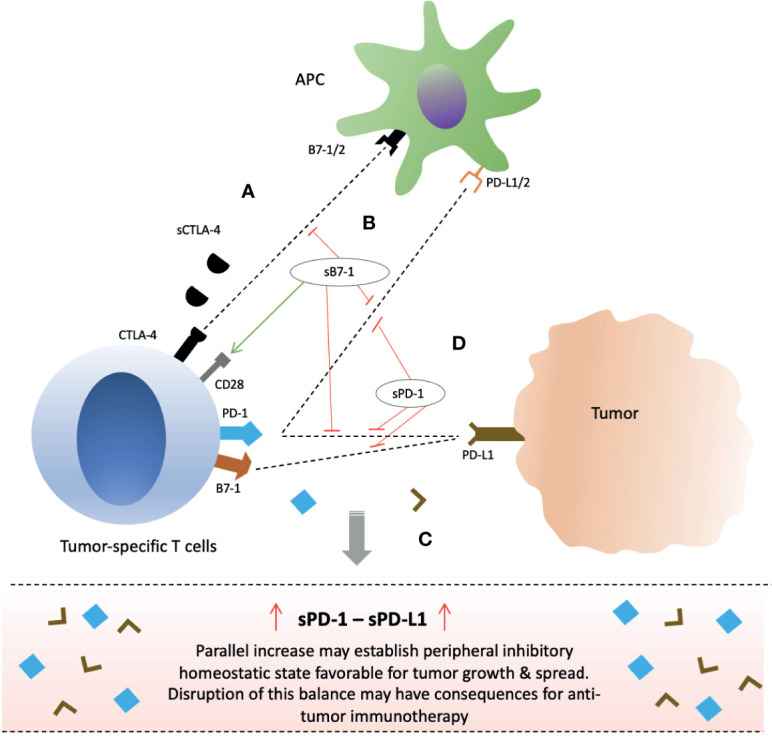
Clinical significance of soluble forms of CTLA-4 and PD-1 checkpoint molecules and their potential for anti-cancer immunotherapy. **(A)** sCTLA-4 exert its inhibitory effects *via* CD28 antagonism. Blockade with anti-CTLA-4 monoclonal antibody or B7-1 upregulation/gene delivery of sB7-1 may enhance T cell immune responses. **(B)** Gene delivery of soluble B7 increase T cell activity not only through increased CD28 co-stimulation but also simultaneous inhibition of PD-1 and PD-L1 interactions. **(C)** Parallel secretion of sPD-1 and sPD-L1 by tumor-specific T cells and tumor/peritumor tissue, respectively, maintain an inhibitory homeostatic microenvironment in tumor local milieu and peripheral circulation. **(D)** sPD-1 delivery in local milieu through gene therapy may enhance anti-cancer immunity by preventing mPD-1 from interactions with sPD-L1 and mPD-L1/2 present on tumor cells or APCs, and also blocking PD-L1 and B7-1 interactions. Abbreviations: CTLA-4; PD-1; sCTLA-4, PD-L1, APCs.

Secretion of soluble CTLA-4 is increased upon T cell activation in response to antigen but comparatively lower in proportion to fl-CTLA-4 and with distinct peak timings (71–74). sCTLA-4, similar to fl-CTLA-4, is discovered to play inhibitory role by binding to B7 ligands on APCs (40, 71). *In vitro* blockade of sCTLA-4 in humans with isoform-specific antibodies was demonstrated to reverse T cell inhibition, and increase both Ag-driven proliferation of T cells (CD4+ & CD8+ T cells) and cytokine production (IFN-γ & IL-17) (40, 71). *In vitro* murine T cells were also shown to produce sCTLA-4 in response to antigen and were inhibited by them (71). Furthermore, *in vivo* blockade of sCTLA-4 was able to protect against metastatic melanoma in mice (71). These outcomes suggest that immune system, in addition to flCTLA-4, may also utilize its soluble form in regulation of T cells, and that it may partially be responsible for the inhibitory effects observed with membrane-bound CTLA-4 (mCTLA-4) molecules (5, 73). It is also in coherence with mechanism of T cell inhibition by mCTLA-4 which is not only dependent on direct intracellular signaling but also through competitive antagonism of CD28 (76). Therefore, soluble CTLA-4 molecules may exert their inhibitory function mainly through competition against CD28 molecules (**Figure 3A**). As such, its blockade with anti-sCTLA-4 mAb may hold potential for anti-cancer therapy.

Soluble CTLA-4 can be detected in the sera of healthy individuals (40, 68). Increased serum levels are detected in several autoimmune diseases and cancer (29–32, 77–86). Higher serum levels of sCTLA-4 was shown to reflect a state of active disease and progression (77–81). Significant elevation in the soluble form of CTLA-4 was detected in 70% of B-cell acute lymphoblastic leukemia (B-ALL) pediatric patients (82). Moreover, elevated serum levels were positively correlated with percentage of leukemic B lymphocytes and sCTLA-4 transcript expression in B cells (82). In patients with prostate cancer, sCTLA-4 levels were significantly associated with biochemical recurrence and progression risks (31). Studies have also shown an increase in sCTLA-4 levels after treatment which may or may not have an impact on prognosis (77–80). sCTLA-4 level was significantly increased 2.64-fold after 2 weeks of sorafenib treatment in HCC patients (29). Similar increase was also shown at week 1 after TACE (trans-arterial chemoembolization) induction (30). Though, effect of this increase on prognosis was not evaluated in both studies (29, 30). In a study of 141 advanced cancer patients (lung cancer, esophageal cancer, liver cancer, ovarian cancer & cervical cancer), higher sCTLA-4 serum levels after treatment induction were significant for longer progression free survival and overall survival in all treatment groups that included radiotherapy (RT) group, chemotherapy group, chemoradiotherapy (CRT) and overall patient groups (83). A negative correlation was observed for sCTLA-4 levels after treatment induction with tumor node metastasis and lymph node metastasis (83). In this case, elevated sCTLA-4 levels after treatment may reflect re-activation of tumor-specific T lymphocytes through increased antigen presentation resulting in improved outcome. On the other hand, elevated levels of sCTLA-4 in serum and pleural effusion before therapy was associated with 20% and 60% decrease in death rates in patients with malignant pleural mesothelioma (MPM) (84). Furthermore, ipilimumab treated melanoma patients with higher serum CTLA-4 levels at baseline were associated with best overall responses and improved overall survival advantage (85, 86). These patients were also at higher risk for immune-related adverse events (86). As such, sCTLA-4 may serve as a prognostic biomarker for anti-CTLA-4 blockade immunotherapy. Moreover, addition of anti-sCTLA-4 specific antibodies may show synergism in inducing anti-cancer responses. In summary, elevated baseline sCTLA-4 levels reflect an immunosuppressive environment which may undergo dynamic changes depending on the treatment induction with predictive and prognostic implications. sCTLA-4 is largely unexplored in cancers and its further evaluation may bolster some of these points.

### Soluble B7-1/B7-2

Both ligands, B7-1 (CD80) and B7-2 (CD86), are recognized by CD28 resulting in T cell activation. CTLA-4 recognizes these ligands with greater affinity and avidity than CD28 causing T cell inhibition (87, 88). Though considered rather similar ligands, they are distinct from each other in their expression profiles and their response to CTLA-4 ligation (89–92). B7-1 expression is induced after activation while B7-2 is constitutively expressed on APCs and is upregulated upon activation (87–92, 95–260, 262, 263). Similarly, the binding ratio of CTLA-4 to B7-1 (20 to1) and B7-2 (8 to 1) is greater than CD28 (93, 94). Consistent crystallographic data have shown a lattice like structure formed as a result of CTLA-4 homodimers interaction with alternating B7-1 homodimers (89). Moreover, at the immunological synapse, B7-1 preferentially recruits CTLA-4 while B7-2 stabilizes CD28 (95). Based on such data, B7-1 is predominantly considered a CTLA-4 ligand while B7-2 as a predominant CD28 ligand. These dynamics will be important to keep in mind while assessing the role of soluble forms of these ligands.

### Soluble B7-1

A soluble form of B7-1 can be detected in the sera of healthy individuals and is expressed by unstimulated B cells and monocytes as well as activated T and B cells (96, 97). Soluble B7-1 (sB7-1) is generated by alternative splicing of B7-1 mRNA with the exclusion of exon encoding for the transmembrane region (96, 97). Similar to mB7-1, sB7-1 structural analysis have revealed that it may also exist as a homodimer (97–99). Soluble B7-1 have shown the ability to bind to its receptors; CD28 and CTLA-4 on T cells. Moreover, sB7-1 has also demonstrated inhibition of mixed lymphocyte reaction (MLRs), T cell proliferation and IL-2 production (96, 97). These outcomes suggest a preferential CTLA-4 binding for soluble B7-1/immunoglobulin G fusion protein (sB7-IgG) and inhibiting T cells functions. Elevated sB7-1 levels detected in certain cancers such as chronic lymphocytic leukemia (CLL), mantle cell lymphoma (MCL), and hepatocellular carcinoma (HCC), and its association with poor prognosis also emphasize its inhibitory role (30, 98). However, increasing data in this direction suggests sB7-1 may prompt stimulatory effect in T cells resulting in improved anti-tumor immunity against tumor cells (100–105). Preincubation of leukemic cell line (WEHI-3 cells overexpressing CD32) with sB7-IgG could increase B7 molecules density on its membrane which in the presence of first signal could enhance T cell activation, cytotoxicity, and IL-2 secretion by activated mouse T lymphocytes (100) (**Figure 3B**). *In vivo* complete regression of established tumors in tumor bearing mice and improved survival was achieved after being administered with therapeutic administration of sB7-IgG (101). Soluble B7-IgG mediated tumor rejection was CD8+ T cell dependent and IFN-γ independent. Furthermore, memory responses were also generated (101). Soluble B7-IgG preferential binding to CD28 T cells or binding to CTLA-4 thereby blocking negative signals and indirectly improving the co-stimulation may explain these outcomes. Besides, *in vitro* and *in vivo* studies have demonstrated that soluble B7-1 may also interrupt PD-1/PD-L1 interaction thereby inhibiting PD-1 mediated suppression while concurrently activating T cells through CD28 (102–105). Soluble form of B7-1 (B7-1-Fc) could bind PD-L1 on human and mouse PD-L1+ tumor cells and block PD-1 interaction with subsequent T cell activation (102). B7-1-Fc generated sustained IFN-γ production by PD-1+ activated T cells more effectively than antibodies to either PD-1 or PD-L1 (104). *In vivo* murine tumor survival extension by sB7-1-Fc was more effective than PD-L1 antibodies (104). Blocking CD28 with antibodies on human T cells or using T cells from PD1−/− and CD28−/− mice showed that sB7-1-mediated anti-cancer immunity was not only dependent on neutralizing PD-1/PD-L1-mediated immune suppression but also a simultaneous B7-1-CD28 co-stimulation (103–105). B7-1-Fc also delayed tumor growth and promoted tumor infiltration of T cells with *in vivo* treatment of established syngeneic PD-L1+ colon and melanoma tumor models (105). Soluble B7-1 anti-tumor effects were mainly mediated by the activation of downstream signaling components of the CD28 and T cell receptor pathways including EGR1–4, NF-κB and MAPK (105). Furthermore, B7-1-Fc binding to CTLA-4 on activated human PBMC did not suppress the T cell activation as addition of anti-CTLA-4 antibodies could not result in any increase of T cell activation as measured by IFN-γ. It was speculated by the authors that CTLA4 acts as a decoy receptor for B7-1, rather than functioning as a suppressive signaling receptor (105). Nonetheless, recent developments of discovering *cis* and *trans* interaction between PD-L1 and B7-1 may explain the efficacy of sB7-1.

A third dimension to the interaction between these two pathways has emerged in which B7-1 and PD-L1 binds in *cis* on APCs and in *trans* between T cells and tumor cells (106–110). Understanding these mechanistic intricacies may also explain the aforementioned efficacy of soluble B7-1. For example, results of a recent study indicate that blockade of PD-L1 interaction with B7-1 in *trans* can augment tumor immunity *via* down-regulating the effect of PD-L1 interaction with PD-1 that also required CD28-dependent activation (107). This result resembles the ones obtained with the use of soluble B7-1. Moreover, such a scenario also resembles the PD-L1/B7-1 cis-interaction on APCs reported in recent studies (108–110). In the tumor microenvironment, PD-L1/B7-1 cis-interaction increases or decreases T cell activation depending on the respective abundance of PD-L1 *versus* B7-1. Abundant B7-1 compared to PD-L1 reduces PD-L1/B7-1 cis-interaction and increases free B7-1 which consequently leads to improved T cell activation. In such a case, both the free B7-1 and the PD-L1/B7-1 cis-heterodimer bind to CD28 to induce a co-stimulatory signal while decreasing the PD-1/PD-L1 binding (108–110). Cis-interaction between PD-L1 and B7-1 has prompted new reconsiderations into already established immune checkpoint blockade immunotherapies (111). Thus, it can be concluded that effects of sB7-1 may vary according to the context. In lymphoid tissues where priming of T cells occurs, increased levels of sB7-1 may actually interact with CTLA-4 to promote T cell inhibition. It may also constitute the predominant source of circulating sB7-1 levels observed in cancers such as melanoma. On the other hand, in the tumor microenvironment where B7-1 and B7-2 are predominantly expressed on APCs may interact in *cis* and *trans* with PD-L1 expressed on tumor cells, the effects of sB7-1 are observed as stimulatory on T cell function.

Soluble B7-IgG as an adjuvant have also shown to enhance the anti-tumor effects of other cancer therapies. Mixing of B7-IgG as a vaccine adjuvant with irradiated tumor cells yielded a stronger *in vitro* anti-tumor activity as compared to alone (101). Intramuscular gene transfer of B7-IgG fusion gene also had induced potent anti-tumor immunity as an adjuvant for DNA vaccination (112). *In situ* expression of soluble B7-1 in the context of oncolytic herpes simplex virus also induced effective anti-cancer immunity (113). Oncolytic herpes simplex virus vectors armed with soluble B7-1, IL-12, and IL-12 resulted in highest efficacy as compared to each alone or the combination of two (114, 115). Moreover, intravenous injection of fusion protein combined with regulatory T cell depletion had caused complete regression of solid tumors with generation of immunologic memory (116). In fact, in murine leukemia and lymphoma models, intravenous administration of fusion protein combined with conventional chemotherapy could induce curative T cell dependent antitumor responses and immunologic memory (117).

Association of elevation sB7-1 levels with prognosis and its dynamics during the treatment has not been well investigated. Elevated levels were observed in CLL, MCL, clear cell renal cell carcinoma (ccRCC), prostate cancer and HCC patients (29–32, 98). Increased levels in CLL were associated with poor prognosis (98). Significant correlation was revealed for sB7-1 levels with biochemical recurrence and progression risk in prostate cancer patients (32). In HCC patients, sB7-1 serum levels were observed to decrease significantly at week 1 after sorafenib treatment; however, an increase in sB7-1 levels at week 2 and 4 was discovered (29). An increase in serum levels at week 1 was also observed for HCC patients after receiving TACE (trans-arterial chemoembolization) (30). No association for sB7-1 levels with prognosis was demonstrated in these studies (29–32). Therefore, sB7-1 levels as predictive and prognostic biomarker and the significance of its dynamics upon treatment induction is not well established and would need further exploration in this context.

### Soluble B7-2

There are not many studies reporting prognosis and clinical significance of soluble B7-2 in cancer patients. Soluble form is generated through B7-2△TM mRNA translation, expressed by resting monocytes, dendritic cells and certain cancer cells (acute lymphocytic leukemia; AML and B-cell chronic lymphocytic leukemia; B-CLL), and can be detected in the sera of healthy individuals (41, 118). Autoimmune disease and cancer have reported elevated levels of sB7-2 (118–122). Recombinant B7-2△TM binds to CD28 and CTLA-4 and induce the T cells activation after stimulation with anti-CD3 mAb (41). While stimulation with Flu M1 peptide could generate IFN-γ production by virus-specific CD8+ memory human T cells (41). *In vivo* therapeutic efficacy of B7.2-IgG was also demonstrated in several tumor models with complete regression of established tumor and increased survival of tumor-bearing mice (101). The anti-tumor responses shown by B7.2-IgG were CD8 dependent and similar to that of B7.1-IgG in all tumor models (101). However, an inhibitory role of sB7-2 was suggested *via* binding to CTLA-4 and transferring negative signal to T lymphocytes as co-delivery of sB7-2 had downregulated the immune response to a DNA vaccine (123). It appears that soluble B7-2 also assumes a stimulatory role as it has been termed stabilizer of CD28 due to its comparative preference for CD28. Moreover, unlike B7-1, B7-2 does not interact with PD-L1 in cis on APCs. Therefore, both ligands improve anti-cancer immunity with distinct underlying mechanisms when introduced into tumor microenvironment in soluble forms. Its inhibitory role; however, would need further exploration.

Elevated levels of sB7-2 could be found in a proportion of leukemia (AML, B-CLL) and HCC patients (29, 30, 118). Significantly elevated levels in 10/24 AML patients were detected at presentation or relapse but patients in remission (n=6) contained only low levels of sB7-2 (118). Similarly, a quarter of AML and myelodysplastic syndrome (MDS) patients had elevated levels of sB7-2 compared to normal healthy individuals but only AML patients with higher sB7-2 levels were associated with lower complete remission (CR) rates and poorer survival in comparison to AML patients with normal sB7-2 levels (121). These outcomes suggest sB7-2 play an inhibitory role in modulating mB7-2 signalling during the malignant process, and represents an independent prognostic marker. Myeloma patients also had significantly elevated levels of sB7-2 but univariate analysis revealed an association of elevated sB7-2 levels with significantly shorter (P < 0.001) survival (median = 22 *vs.* 51 months) and event-free survival (median = 14 *vs*. 31 months) only in the treatment arm receiving ABCM + P (adriamycin, armustine, cyclophosphamide, and melphalan with prednisolone) and not ABCM patients (122). HCC patients had also observed a significant increase in sB7-2 levels at week 4 of the sorafenib treatment but no association was sought (29). More studies would be required to establish its role as a predictive and prognostic biomarker in cancer.

## PD-1 Inhibitory Checkpoint Molecules Axis

The PD-1/PD-L1/PD-L2 checkpoint pathway plays a critical role in regulation of T lymphocytes in cancer and its disruption has been manifested in the improved clinical outcome for cancer patients (7–10, 15–21). Several studies have shown elevated levels of soluble forms of these checkpoint protein molecules in the sera of cancer patients which has excellently correlated with poor prognosis, particularly the sPD-L1 (29, 30, 124–143). Moreover, a positive correlation has been reported in several cancers between the plasma levels of sPD-1 and sPD-L1 (29, 30, 124, 125, 137). Such positive correlation may suggest a common provenience, simultaneous secretion, and regulatory interplay in the same manner as their membrane-bound counterparts (29, 30, 124, 125, 137). As such, secretion of soluble forms may represent an attempt for tumor invasion and spread as elevated sPD-L1 levels have been correlated with advanced disease and metastatic status in various cancers (129, 135, 136, 138–141). As a homeostatic peripheral tumor immune evasive environment may prevail favoring tumor growth (**Figure 3C**). Much is the same way; disruption of this balance may also have implications for cancer immunotherapy. In fact, post-therapeutic increase in sPD-1 and reduction in sPD-L1 have been associated with improved outcome (128, 141–147). We will further elucidate these implications in the context of each soluble molecule alone – sPD-1 and sPD-L1.

### Soluble PD-1

Programmed cell Death-1 (PD-1) protein is a type I transmembrane glycoprotein and is expressed on T cells after its activation (21, 148). In addition, other immune cells also exhibit PD-1 expression including B cells, NK cells, NKT cells, APCs, innate lymphoid cells (ILC2) and other myeloid cells (50, 55, 148–151). PD-1 ligation to its ligands, PD-L1 and PD-L2 expressed on tumor cells or APCs, results in T cell inhibition and immune escape, and its blockade with monoclonal antibodies have yielded excellent clinical outcome (21, 50, 55, 148). A soluble form of PD-1 can be detected in the plasma of healthy individuals and elevated levels are found in autoimmune diseases, chronic infections and various cancers (124–126, 137, 141–144, 152–156). Several solid and hematologic cancers that have shown elevated sPD-1 levels include non-small cell lung carcinoma (NSCLC), HCC, nasopharyngeal carcinoma (NPC), pancreatic adenocarcinoma, advanced rectal cancer, metastatic melanoma, diffuse large B- cell lymphoma (DLBCL), and CLL (124–126, 137, 141–144, 152–156). Soluble form is produced through alternative slicing of full-length PD-1 transcript which is composed of five exons (152). The resulting four splice variants lacks single or combination of the middle exons 2, 3, and 4. Only one splice variant that lacks exon 3 (PD-1 △x3) but retains other exons (1, 2, 4, 5) may encode for soluble form of PD-1 (152). *In vitro* activation of T cells has shown to produce soluble PD-1 (152). Tumor site and tumor-specific T cells may constitute the primary source of circulating sPD-1 as reduction of HCC was shown to cause a decrease in circulating sPD-1 levels (126). In a separate study, circulating tumor-specific T cells were identified to be the prime source of sPD-1 as absent tumor-infiltrating lymphocytes (TILs) in melanoma patients was associated with high sPD-1 levels in plasma as opposed to brisk (TILs present across the entire base of the tumor) or non-brisk (TILs distributed only focally) TILs (153).

Dynamics of soluble PD-1 has demonstrated variation in clinical significance before and after induction of treatment. Pretherapeutic elevated plasma levels of sPD-1 correlates with disease status, disease activity (severity and progression) and in some cases with prognosis (124–126, 137, 141–144, 152–156). It has been associated with systemic inflammation markers (CRP) in advanced pancreatic cancer (124, 137), viral load, viral activity and HCC risk in HBV patients (126, 155), and worst prognostic indicators in DLBCL patients (156). However, it has not been well correlated with prognosis in these cases. Only two studies have shown its correlation with worst prognosis that involved pancreatic adenocarcinoma and HBV-related HCC patients (124, 126). On the other hand, any increase in sPD-1 levels after therapy has been correlated with improved outcome in several studies (125, 141–143). Improved progression free survival and overall survival was associated with increased sPD-1 levels after EGFR TKIs treatment in NSCLC patients (143). Increase in sPD-1 after receiving two cycles of nivolumab was also correlated with improved outcome for NSCLC patients in terms of PFS and OS (143). In NPC patients, delivery of IMRT had led to an increase in sPD-1 which was associated with decreased plasma EBV-DNA level and improved survival (142). These outcomes imply that pretherapeutic elevated sPD-1 may signifies disease severity and immune tolerance as it is observed in the case of mPD-1. While its increase after treatment induction; however, may indicate re-activation of immune responses against tumor antigens. For example, radiation therapy is believed to induce tumor-specific immune responses because of surge in antigen presentation by APCs (157, 158). EGFR TKIs, as well, has been revealed to up-regulate HLA-I which is associated with tumor-specific CD8+ T lymphocytes mediated immunity (159, 160). These tumor-specific T cells may actually constitute the source of observed increase in sPD-1 after such treatments. In broad, sPD-1 levels poorly correlate with prognosis probably due to the dynamic changes incurred in sPD-1 levels after treatment induction. Hence, changes in sPD-1 levels after treatment may serve a better predictor and prognostic indicator than baseline levels.

Activation of human PMBCs with anti-CD3 plus CD28 mAbs has demonstrated a parallel increase in flPD-1 and PD-1△x3 transcripts suggesting an important interplay between membrane bound and soluble forms in preventing autoimmunity and peripheral self-tolerance (152). Soluble PD-1 is shown to be biologically active and capable of inhibiting mPD-1/PD- L1 and mPD-1/PD-L2 interactions (161–163) (**Figure 3D**). In addition, PD-L1 has also been revealed to cause T cell inhibition in an indirect manner by binding to B7-1 (CD80) thereby disrupting B7-1 and CD28 interactions (106) (**Figure 3D**). Therefore, sPD-1 may serve as an anti-PD-L1 antibody with triple targets: mPD-1/PD- L1; mPD-1/PD-L2; and PD-L1/B7-1. As such, *in vitro* and *in vivo* blockade of PD-L1 and PD-L2 by sPD-1 delivered *via* gene therapy was shown to enhance tumor-specific T cell responses resulting in suppression of tumor growth (161–164). Soluble PD-1 transferred *via* eukaryotic expression plasmid was shown to increase T cell activation, cytotoxicity and tumor reduction *via* blocking of PD-L1 and PD-L2 expressed on tumor cells and/or DCs (161–163). Increased mRNA expression of IFN-γ, TNF-α, 4-1BB and B7-1 with downregulation of OX40 and IL-10 was noticed in the splenocytes (161). Moreover, inhibitory effect of sPD-1 on tumor was similar to that of mice injected with anti-PD-L1 mAb (161). Anti-tumor immunity induced by soluble PD-1 has also been demonstrated in animal studies using reconstructed adeno-associated virus plasmid encoding sPD-1 (164). Tumor regression with tumor-specific T cell infiltration and improved survival was achieved with sPD-1 local delivery (164). Nonetheless, reverse signaling through sPD-1 has also been suggested resulting in inefficient DC maturation (165). Therefore, sPD-1 delivery *via* gene therapy into the local tumor microenvironment holds great potential as therapeutic strategy.

**Figure 4:**
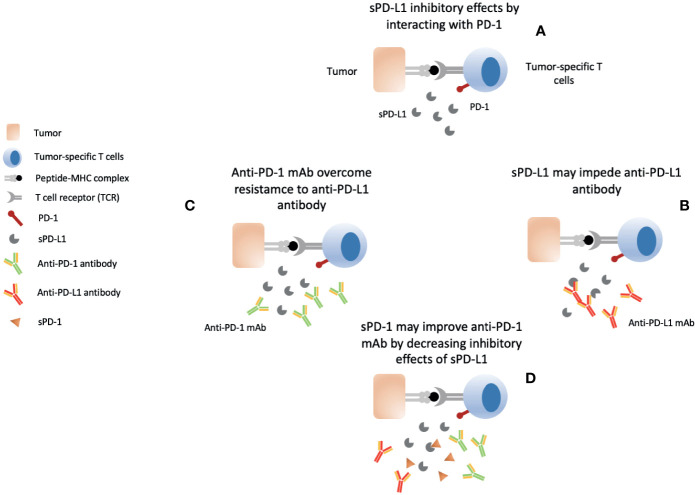
Soluble PD-L1 and Anti-PD-1/PD-L1 mAb immunotherapy conundrum. **(A)** Elevated circulating sPD-L1 in various cancers correlates with prognosis and exerts its inhibitory effects on tumor-specific T cells by interacting with PD-1 receptor. **(B)** sPD-L1 impedes efficacy of anti-PD-L1 antibodies. **(C)** Anti-PD-1 may overcome resistance to anti-PD-L1 antibodies by reducing interaction between sPD-L1 and PD-1 **(D)** sPD-1 may overcome sPD-L1-mediated resistance to anti-PD-L1 antibody by directly interaction with sPD-L1 and mPD-L1. Anti-PD-1 antibody addition may result in a stronger response.

Local gene delivery of sPD-1 can enhances the anti-tumor effects of other local gene therapeutic agents probably *via* reducing the inhibitory effects of PD-1/PD-L1 interaction upregulated after tumor-specific T cells activation (163, 166–174). Soluble PD-1 delivery has successfully improved the cytotoxicity of tumor-specific CTLs induced by the local gene delivery of secondary lymphoid chemokine (CCL21) using eukaryotic expression plasmids (pSLC) (163). Likewise, sPD-1 has also enhanced the anti-cancer immunity induced by CH50 that is a recombinant polypeptide with 2 functional domains (CellI, and HeparinII) for targeting fibronectin which is aberrantly expressed matrix glycoprotein in cancer associated with facilitating tumor growth, invasiveness, metastasis, and resistance to therapy (166, 167). Anti-cancer effects of sPD-1-CH50 included intensification of macrophages and cytotoxic T lymphocytes’ (CTLs) cytotoxic activity through inducible nitric oxide synthase (iNOS), tumor necrosis factor-alpha (TNF-α), IFN-α with demonstration of *in vivo* restriction of hepatoma growth and invasiveness (166). *In vitro* and *in vivo* anti-cancer effects of thymidine kinase expression through adenovirus harboring herpes simplex virus thymidine kinase gene (HSVtk) delivery were further exaggerated by sPD-1 (168). As a vaccine adjuvant, sPD-1 has also demonstrated improvement of vaccine efficacy including heat shock protein 70 (HSP70) vaccine and human papilloma virus-16 E7 DNA vaccines (169–171). Combined gene expression of 4-1BB ligand and sPD-1 could enhance CD8+ T cells infiltration, greater tumor growth inhibition and improvement in survival of tumor bearing mice (172). Furthermore, it had also been able to enhance the T cell and NK cell immunity induced by IL-21 through blocking the PD-1/PD-L1 interaction pathway (173). Ultrasound-mediated co-delivery of sPD-1 and miR-34a which is implicated in PD-L1 upregulation had induced tumor apoptosis with increased IFN-γ secretion and percentage of CTL (174). In this sense, PD-L1 regulators could be targeted with combination of sPD-1 to overcome resistance and improve tumor-specific immunity (175). Furthermore, conventional anti-cancer therapies such as radiotherapy and chemotherapy have also been associated with induction of anti-cancer immunity and consequent PD-L1 upregulation which could be countered by sPD-1 (176–181). Association of circulating soluble PD-L1 with patients receiving chemotherapy and radiotherapy also indicates their candidacy for sPD-1 delivery in order to reduce the inhibitory effects of sPD-L1 (182–184). sPD-L1 levels have also shown resistance to anti-PD-L1 monoclonal antibody immunotherapy which was overcome by anti-PD-1 mAb therapy (185–189) (**Figure 4**). Likewise, addition of sPD-1 may also overcome resistance to anti-PD-L1 mAb as anti-PD-1 mAb shares the same targets with sPD-1 and both have shown similar *in vitro* anti-cancer immunity (161, 185). As such, sPD-1 may be used as alternative to anti-PD-1 mAb or its addition may further enhance the activity of anti-PD-1 mAb and reduce the dosage needed for optimum efficacy which may further reduce the risk of adverse events associated with mAb-based immunotherapy (185). Hence, sPD-1 represents an excellent candidate for exploitation as therapeutic strategy in cancer patients as single agent, to complement immunotherapy and other traditional cancer treatments, and as an adjuvant with gene therapies and vaccines.

### Soluble PD-L1

In comparison to sPD-1, soluble form of its ligand - sPD-L1, has been extensively investigated for its clinical significance. It can be detected in the plasma of normal individuals but elevated levels are reported in autoimmune diseases and cancer (126–128, 190–194). Soluble form can be produced by proteolytic cleavage as sPD-L1 was only detectable in supernatants of mPD-L1+ cell lines which was suppressed by MMP inhibitors implicating matrix metalloproteinase activity (195). Cytokines such as IL-21 and IL-6 may also mediate its release as its secretion was increased in the culture media of Waldenstrom macroglobulinemia (WM) cell lines with these cytokines (193). In addition, recent studies have also described two distinct types of splicing events affecting or removing exon encoding for transmembrane domain (185, 189, 196–199). Tumor cells and mature DCs were identified as the main sources of sPD-L1 (127, 194, 200). Nonetheless, other cells in the tumor stroma may also be responsible for circulating elevated sPD-L1 levels as discrepancies have been observed between tumor expression of mPD-L1 and circulating sPD-L1 (125, 128, 137, 201). In addition, recent studies have also identified cell-free PD-L1 in exosomes (exPD-L1) which is membrane-bound and may confound the detection of membrane-free sPD-L1 (202–204).

Soluble PD-L1 in plasma is believed to retain its binding capacity and inhibitory properties (45, 127). *In vitro* studies have suggested an inhibitory role for sPD-L1 demonstrating its ability to suppress T cell activation, reduce secretion of IFN-γ and induction of T cell apoptosis (127, 189, 193, 196, 197, 200). Tumor cells as well as DC-released sPD-L1 has demonstrated the ability to induce *in vitro* T cell apoptosis (127). Soluble PD-L1 and PD-L2 secreted by WM cells could reduce T cell proliferation which was associated with a decrease in the cell cycle protein cyclin A, phosphorylated Akt (p-Akt) and p-ERK protein levels. T cell metabolic rate was also altered *via* reducing mitochondrial adenosine triphosphate production and maximal respiratory capacity (193). Nonetheless, studies have also attributed these immune inhibitory properties of cell-free PD-L1 to exPD-L1 in melanoma, glioblastoma, and mouse models (202–204). Moreover, *in vitro* and patient studies have also reported sPD-L1 completely lacking such inhibitory properties (185, 202). Ng, et al. revealed sPD-L1 rather functions as a receptor antagonist blocking the inhibitory function of membrane-bound PD-L1 expressed on cellular or exosomal membranes (199). Hence, further evaluation of sPD-L1 in terms of its primary source and its course of action would further be needed to establish its role as an inhibitory soluble receptor.

Discovery of PD-L1 on exosomes has been regarded as the alternative mechanism for immunosuppression activity of this checkpoint. Systemically introduced exosomal PD-L1 or secreted by tumor cells have shown the capability to bind to PD-1, induce T cell inhibition, and promote tumor growth (202–204). Exosomal PD-L1 was also able to induce suppression of T cell activation in draining lymph nodes, and its genetic blockade was shown to inhibit tumor growth, and promote T cell activity in the draining lymph node to induce systemic anti-tumor immunity and memory (203). Exosomal PD-L1 level was upregulated by IFN-γ which suppressed the CD8+ T cells and facilitated the tumor growth (202, 204). Circulating levels of exPD-L1 and IFN-γ were positively correlated in metastatic melanoma patients (202). In glioblastoma patients, PD-L1 DNA was found in circulating extracellular vesicles (EVs) which was correlated with tumor volumes of up to 60 cm^3^ (204). Exosomal PD-L1 appears to be resistant to anti-PD-L1 but not anti-PD-1 antibody blockade (202–204). In fact, blockade of exPD-L1 was shown to work additively with anti-PD-L1 antibodies to suppress tumor growth (203). Furthermore, removal of exPD-L1 was shown to inhibit tumor growth, even in models resistant to anti-PD-L1 antibodies (203). On the other hand, anti-PD-1 blockade could successfully reverse the exPD-L1-mediated T cell inhibition in melanoma and glioblastoma (202, 204). In metastatic melanoma patients, changes had occurred in circulating exPD-L1 levels during the course of anti-PD-1 therapy (202). Stratification of clinical responders and non-responders could be achieved based on the magnitude of early on-treatment increase in circulating exPD-L1 which indicated adaptive response of tumor cells to re-invigorated T cells (202). Understanding the mechanistic details of exPD-L1 provides a rationale for its application as a prognostic biomarker and predictor of response to anti-PD-1 therapy.

Various cancers including NSCLC (129, 138, 205), RCC (127), DLBCL (128, 130), oral squamous cell carcinoma (OSCC) (139), multiple myeloma (MM) (131), nasal NK/T cell lymphoma (NKTTL) (132), papillary thyroid cancer (PTC) (133), epithelial ovarian cancer (EOC) (134), gastric cancer (135, 140, 206), HCC (136), and WM (193) have demonstrated significantly higher circulating levels of sPD-L1 in comparison to healthy individuals. In several of these studies, sPD-L1 level was significantly associated with certain cancer attributes such as: clinical stage, tumor cell differentiation, and lymph node status in OSCC (139) and gastric cancer (135, 140); abdominal organ metastases, cancer histopathology (adenocarcinoma) in NSCLC (129, 138); tumor size, stage and grade, and tumors with necrosis in RCC (127); stage of cirrhosis and stage of HCC in HCC (136); residual tumor burden in EOC (134); and extrathyroidal extension in PTC (133). Moreover, cancer patients with higher sPD-L1 level had revealed significant shorter OS, PFS and higher mortality rates (127–136). Such outcomes suggest an inhibitory role for sPD-L1, as well as, reveal a consistent outlook for sPD-L1 association with advance disease and worst prognostic factors which indicate its role as a strong predictive and prognostic biomarker.

Presence of elevated plasma level of PD-L1 has been shown to determine the response of various anti-cancer treatments including surgical reduction, chemotherapy, radiotherapy, anti-EGFR treatment, and anti-PD-1 immunotherapy (131, 207). MM patients with low sPD-L1 had reported a better response to treatment that consisted of a mixture of treatment regimens including novel drugs such as bortezomib and lenalidomide (131). Similarly, sPD-L1 levels in the bone marrow plasma of MM patients also predicted the progression of autologous transplantation (207). The clinical benefit by inhibition of PD-1 therapy was significantly associated with baseline serum PD-L1 levels in NSCLC and metastatic melanoma (186, 187). Pre-chemotherapy levels were associated with overall survival in advanced lung cancer, advanced gastric cancer, biliary tract cancer, and DLBCL (128, 130, 145, 184, 208, 209). In epithelial ovarian cancer patients, soluble PD-L1 did not only predict prognosis but was also correlated with platinum response (134). Response to concurrent chemoradiotherapy or radiotherapy alone was also associated with sPD-L1 circulating levels in NSCLC, HCC, and NNKTL patients (132, 182, 183). Prognosis was predicted based on high levels of sPD-L1 in HCC patients receiving surgical reduction, local ablation, sorafenib and liver transplantation (125, 136). Gastric cancer patients receiving surgical reduction with high sPD-L1 levels had better prognosis and lower recurrence (135). As such, identification of baseline sPD-L1 levels may serve as a great predictive of response to various treatments, and as a prognostic marker as well.

Soluble PD-L1 levels increase or decrease after undertaking various anti-cancer treatments may hold predictive and prognostic significance. NSCLC patients receiving TRT showed a significant decrease in sPD-L1 at week 2 and week 4 compared to baseline levels; however, sPD-L1 levels returned to baseline levels post-TRT (182). Nonetheless, this decrease in sPD-L1 was not evaluated for prognostic significance. A contrast result was observed in patients with locally advanced rectal cancer treated with neoadjuvant chemoradiotherapy (144). Soluble PD-L1 levels were significantly increased after CRT (p < 0.0001) and high sPD-L1 level after CRT tended to be associated with worse DFS (p = 0.0752). Likewise, hepatocellular carcinoma patients receiving concurrent chemoradiotherapy displayed increased sPD-L1 levels post-RT which decreased back to baseline levels at 1 month while SBRT receiving patients exhibited a continued increase until 1 month (183). The pattern of sPD-L1 change over time was significantly different between the two groups but their prognostic significance was not sought. Radiation therapy is known to re-invigorate tumor-specific T cells which may cause an early decrease in sPD-L1; however, tumor cells upregulation of PD-L1 in response to re-invigorated T cell may suggest the afterward increase in sPD-L1 levels. Further exploration of such dynamics and their significance with prognosis must be sought in order to fully appreciate sPD-L1 potential as a biomarker.

Dynamics of sPD-L1 in cancer patients receiving chemotherapy have rather presented a persistent outlook. Baseline sPD-L1 level was well correlated with prognosis in cancer patients receiving chemotherapy (128, 145, 146, 184). At disease progression, sPD-L1 levels were significantly increased in comparison to baseline levels (145, 184). A trend of inverse relationship between sPD-L1 and tumor burden in response group was identified in biliary tract cancer (BTC) patients (184). Park, et al. further revealed that patients whose sPDL1 increased after 1st cycle of chemotherapy showed the tendency of worse PFS and OS (145). DLBCL patients had also shown a significant decrease in sPD-L1 levels at complete remission or at the end of the treatment as compared to sPD-L1 levels at diagnosis (128, 146). Patients with complete response had achieved normal levels of sPD-L1 as were observed in controls (146). Therefore, baseline sPD-L1 levels may predict response to chemotherapy and its increase after chemotherapy indicate prognostic outcome. As such, sPD-L1 levels can be applied as a good predictor and prognostic biomarker in these patients.

NSCLC patients with EGFR mutation had higher sPD-L1 levels compared to wild type and a post-therapeutic significant reduction in sPD-L1 level was only observed in EGFR mutated patients (210). In a separate study, NSCLC patients receiving anti-EGFR treatment showed a median 19.19% change in the pre-treatment and on-treatment sPD-L1 level; though, there was no differences in the treatment response or progression free survival between patients with or without a reduction of sPD-L1 levels (211). Further exploration would be required to identify the cause of reduction in sPD-L1 levels after EGFR treatment and to establish its association with cancer prognosis. Immune checkpoint inhibitors had induced a significant increase in sPD-L1 concentrations at first restaging after 7 to 8 weeks; yet, no prognostic significance was sought (212). In a separate study involving the use of nivolumab (anti-PD-1 mAb) only revealed a similar increase in sPD-L1 levels at first tumor evaluation which was associated with poor response (ORR 17% *versus* 68%, p=0.005), clinical benefit (10% *versus* 47%, p=0.049), shorter median PFS (1.8 months *vs*. 6.5 months, p = 0.008), and shorter median OS (5.4 months *vs*. NR, p = 0.028) (147). In fact, magnitudes of early on-treatment increase in circulating exPD-L1 after anti-PD-1 therapy in melanoma patients was also shown to stratify clinical responders from non-responders (202). Hence, increase in circulating PD-L1 levels after anti-PD-1 therapy indicates adaptive response of tumor cells to re-invigorated T cells and could be used as a predictor for anti-PD-1 therapy.

## Soluble B7-H3

B7-H3 (B7 homolog 3 protein), a B7 ligand molecule for which the receptor is yet unknown, belong to B7-CD28 family and exhibits co-stimulatory and co-inhibitory properties (39, 213–215). Receptor for B7-H3 could be present on NK and T cells as it appears to inhibit both type of cells (39, 213). T cell stimulation is achieved by binding to TLT-2 receptor while binding to unknown receptor results in T, NK and osteoblastic cells inhibition (213). B7-H3 expression in normal tissues is limited but aberrant expression is reported in a variety of cancers which is also associated with poor outcome (216–218). Overexpressing cancers include renal cell carcinoma, breast cancer, lung cancer, osteosarcoma, neuroblastoma, prostate cancer, esophageal squamous cancer, gastric cancer, pancreatic cancer, gallbladder cancer, colorectal cancer, ovarian cancer, cervical cancer, and endometrial cancer (217, 218). Its blockade has been considered for anti-cancer immunotherapy in preclinical as well as clinical trials involving various types of cancers (219, 220). Hence, like other B7 molecules, B7-H3 is also being recognized as an important checkpoint molecule which needs further exploration.

In addition to membrane B7-H3, a soluble isoform of B7-H3 also exists which is produced through alternate splicing of B7-H3 from the 4th intron encoding for a 248 amino acid length of protein termed as spliced sB7-H3 (221). It can be detected in the PMBCs of healthy donors (44). Spliced sB7-H3 gene was predominantly found in hepatoma and peritumor tissues as compared to PMBCs from healthy donors and HCC patients (221). In addition, release of sB7-H3 from T cells, monocytes, and MDDCs (monocytes-derived dendritic cells) have also been demonstrated upon stimulation (44). Several cancer cells lines positive for membrane B7-H3 have shown to release soluble isoform (44, 222). Soluble B7-H3 release was decreased while surface expression of mB7-H3 was increased in A549 and B7-H3/L929 cells with addition of matrix metalloproteinase inhibitor (MMPI) indicating sB7-H3 release is mediated by MPP (44). Correlation of MMP-9 levels with levels of sB7-H3 in medulloblastoma cells (D283 and D425) treated with B7-H3 over-expressive plasmids also implicate MMP activity (223). Conditioned media from miR-29-, and JQ1-treated cells (MYC inhibitor) demonstrated that overexpression of miR-29 and inhibition of MYC can stifle secretion of sB7-H3 (223). In conclusion, release of sB7-H3 may be promoted by both mechanisms including proteolytic shedding as well as alternative splicing.

**Figure 5:**
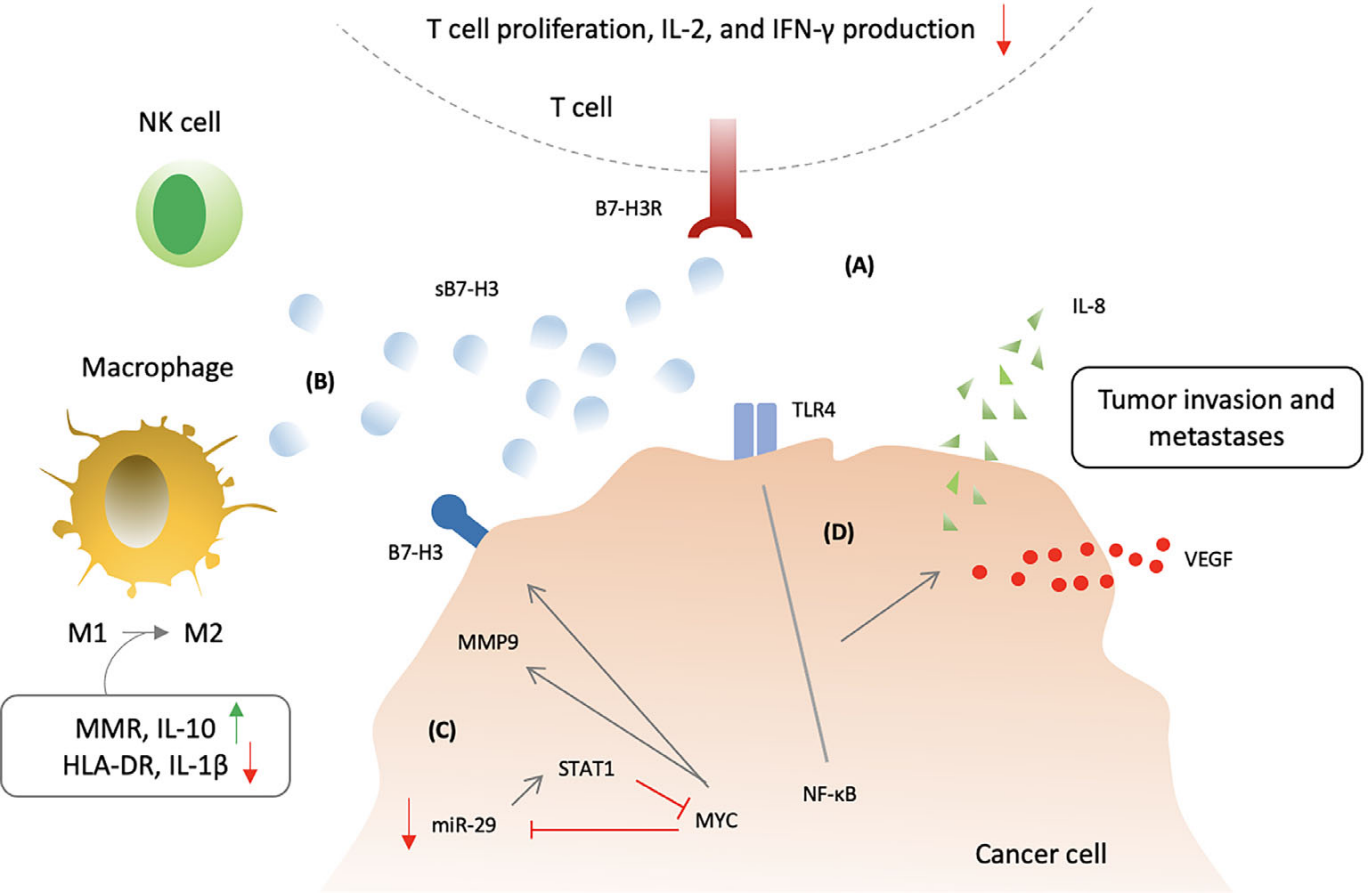
Soluble B7-H3 biological activity and mechanism of action. **(A)** Soluble B7-H3 could disrupt B7-H3:B7-H3R interactions and inhibit T lymphocytes. **(B)** sB7-H3 supress NK cell-mediated tumor cell lysis and also switch macrophage phenotype from M1 (classically activated macrophage) to M2 (alternatively activated macrophage) *via* increasing expression of macrophage mannose receptor (MMR) and IL-10 and decreasing expression of HLA-DR and IL-1β. **(C)** MYC and micR-29 may regulate the secretion of sB7-H3 *via* matrix metalloproteinases 9 (MMP9) as inhibtion of MYC or overexpression of micR-29 could increase the sB7-H3 and MMP9 expression. **(D)** Soluble B7-H3 may also induce cancer invasion and metastases through increased NF-κB activity *via* upregulating toll-like receptor 4 (TLR4) expression with consequent promotion of IL-8 and vascular endothelial growth factor (VEGF) expression.

Soluble B7-H3 is functionally active and retain inhibitory properties (44, 221). sB7-H3 was able to disrupt B7-H3/B7-H3R interactions by binding to B7-H3 receptor present on activated T cells (44) (**Figure 5**). sB7-H3 presence was enough to inhibit T cell proliferation and cause reduction of IL-2 and IFN-γ in the supernatants indicating negative regulation of T cells by sB7-H3 (221). In addition, release of soluble 4IgB7H3 has also shown *in vitro* and *in vivo* suppression of natural killer cell–mediated tumor cell lysis (222). Moreover, sB7-H3 could switch macrophage phenotype from M1 (proinflammatory type; classically activated macrophage) to M2 (anti-inflammatory phenotype; alternatively activated macrophage) through increased expression of macrophage mannose receptor (MMR) and IL-10 and decreased expression of HLA-DR and IL-1β (224). Similar to its surface counterpart, sB7-H3 has also exhibited to promote migration, invasion, metastases and angiogenesis (222, 223, 225). Conditioned media from B7-H3 OE treated cells showed an increase expression of proangiogenic molecules using a human angiogenesis antibody array (222). Higher sB7-H3 levels were evident in B7-H3 OE treated D283 and D425 cells in comparison to control (222). Moreover, levels of sB7-H3 correlated with MMP9 in conditioned media suggesting a possible role for sB7-H3 in angiogenesis through MMP9 modulation (222). In MB cell lines, miR-29-treated cells showed decreased levels of proangiogenic molecules as well as low levels of sB7-H3 (222). MYC inhibitor (JQ1)-treated cells were also associated with low levels of sB7-H3. These outcomes suggest that MYC upregulates sB7-H3, and is inhibited by miR-29 overexpression. Soluble B7-H3 was also shown to increase invasion and metastases of pancreatic carcinoma cells (225). Soluble B7-H3 significantly increased NF-κB activity by upregulating TLR4 expression which promoted IL-8 and VEGF expression, and *in vivo* TLR4-knock-down tumor cells were associated with decreased metastatic ability after being induced by sB7-H3 (225). Interestingly, IL-8 levels along with sB7-H3 were significantly increased in HCC patients which may imply a similar interplay (226).

Soluble B7-H3 can be detected in the sera of healthy individuals (44). Elevated serum levels of soluble B7-H3 in various cancers have been reported and correlated with aggressiveness and prognosis. In NSCLC patients, B7-H3 circulating levels were significantly higher in comparison to patients with obstructive pulmonary diseases and healthy controls (p<0.001) (227). Higher sB7-H3 levels in serum and malignant pleural effusions were correlated with tumor size, stage and metastases (nodal & distant) (228). High grade glioma patients had higher CSF and serum levels of sB7-H3 and sPD-L1 compared to low grade glioma; however, their serum levels in glioma patients did not significantly differ from healthy controls (229). Glioma patients had higher CSF levels of sB7-H3 and B7-H1 as compared to patients with moderate traumatic brain injury (229). Patients with primary HCC revealed to have significantly elevated levels of sB7-H3 in their serum compared to healthy individuals (226). Cirrhotic patients with early-stage HCC (ESHCC) demonstrated significantly higher serum levels than cirrhotic patients (P < 0.001) (230). Moreover, sB7-H3 in cirrhotic patients with ESHCC correlated with tumor size, tumor stage, vascular invasion, and tumor differentiation (230). Bile levels of sB7-H3 in malignant biliary strictures (BS) were reported to be significantly higher than that of benign BS (p<0.001) (231). Tumor stage, vascular invasion, and lymph node and distant metastasis were correlated with sB7-H3 levels. Patients with higher sB7-H3 bile levels reported shorter survival (p=0.014) (231). Osteosarcoma patients also reported significantly higher sB7-H3 levels that correlated with stage, differentiation and metastasis status (p<0.05) (232). Higher levels were also observed in Renal cell carcinoma patients that showed correlation with clinical stage of the cancer (233). In conclusion, sB7-H3 can correlate with advance cancer status and predict poor outcome in various cancer patients. Moreover, as it has shown to promote cancer invasion and metastasis, it can also be exploited for anti-metastatic activity to target cancer spread.

## Soluble B7-H4

B7-H4 is a type I B7 family member and is also known as B7x, B7-S1, or VTCN1. B7-H4 is a novel B7 ligand that can negatively regulate T cell-mediated immune responses (234–236). It can inhibit T cell proliferation, cell-cycle progression, and IL-2 production upon its ligation to yet unknown receptors (234–236). In addition to activated T cells, MDSCs are also reported to express its putative receptor (237). B7-H4 expression is lacking in normal tissues and immune cells. On the other hand, aberrant expression is reported in various cancers which has correlated with poor outcome (14, 39, 238, 239). As such, this checkpoint has shown also potential for immune checkpoint inhibition based immunotherapy (238, 239).

A soluble form of B7-H4 also exist that is generated by proteolytic cleavage mediated by the metalloproteinase activity (240, 241). Just like membrane B7-H4, soluble B7-H4 has also been expressed in a variety of cancers (242–254). Significantly higher serum expression of B7-H4 in gastric cancer (242, 243), NSCLC (244, 245), HCC (246, 247), RCC (248–250), bladder urothelial carcinoma (251), ovarian cancer (252, 253), and osteosarcoma (254) has been reported in comparison to normal healthy individuals or control groups. Soluble B7-H4 levels significantly correlated with tumor size, lymph node metastasis, depth of tumor invasion and TNM classification in gastric cancer, HCC, and RCC (242, 243, 246–248, 250). Additionally, sB7-H4 was also correlated with serum AFP levels in HCC, distant metastasis at nephrectomy in RCC, distant metastasis in osteosarcoma, and histology type in ovarian cancer (247, 248, 250, 252, 254). Soluble B7-H4 levels in malignant pleural effusion were higher in lung cancer and predicted a worst prognosis (255). Elevated preoperative levels had predicted a worse response to anti-VEGF therapy in RCC (250). Furthermore, elevated circulating B7-H4 levels were significantly associated with worst prognosis in gastric cancer, NSCLC, HCC, RCC, and osteosarcoma (242–247, 250, 254, 255). Hence, soluble B7-H4 can be a valuable biomarker for assessing cancer prediction and prognosis in a broad range of cancers.

Function of soluble B7-H4 has not been fully investigated. Its evaluation in mouse models of autoimmune diseases such as rheumatoid arthritis, type I diabetes, and contact hypersensitivity suggested that soluble B7-H4 might block the inhibitory functions of membrane B7-H4 and enhance T-cell-mediated autoimmune responses (240, 256, 257). In contrast, T cell inhibition was demonstrated with B7-H4 wild-type and NLS mutant transfected HEK293 cells that could also produce soluble B7-H4 (241). NLS mutant transfectants which had produced more soluble B7-H4 showed stronger inhibitory effect on T cell proliferation and IL-2 production (241). Furthermore, anti-B7-H4 mAb efficiently blocked the T cell inhibition of supernatants from NLS mutant transfectants (241). Zang, et al. also observed T cell inhibition in the presence B7x-Ig (236). Furthermore, significant elevated serum levels of B7-H4 in cancer patients and its association with invasiveness, progression and worst prognosis also suggests an inhibitory role (242–255). The discrepancy may arise from the its binding to distinct receptors under diverse circumstances as is the case with other B7 family members. Regardless, in case of its inhibitory effects on T cells, its blockade with selective antibodies may serve as an immune checkpoint for anti-cancer immunotherapy exploitation. Nonetheless, further studies are validated to look into the various aspects of soluble B7-H4 regulatory mechanisms and inhibitory effects in cancer.

## BTLA Inhibitory Checkpoint Molecules Axis

B and T lymphocyte attenuator (BTLA, CD272), a novel checkpoint co-inhibitory receptor also belonging to the CD28 superfamily, is constitutively expressed by naïve CD4+ and CD8+ T cells and is upregulated after T cell activation (27, 28, 258, 259). In addition, other immune cells have also shown expression of BTLA such as NK cells, NKT cells, B cells, DCs, and myeloid cells (22, 28, 259). It recognizes HVEM as its ligand which is also expressed on a variety of cells including T cells, B cells, NK cells, DCs, myeloid cells, and is inducible in somatic tissues (22, 259). BTLA ligation to HVEM after T cell activation have shown to inhibit T cell proliferation and effector functions (22–28). Its upregulation in various cancers and association with prognosis suggests that cancers may exploit this pathway for immune evasion as in the case of other T cell co-inhibitory molecules such as CTLA-4 and PD-1 (34, 35). In fact, its blockade has shown enhanced immune responses; as such, BTLA/HVEM has been considered as an emerging new target to enhance anti-tumor immunity (36, 37, 261).

A soluble form of BTLA and HVEM can be detected in the sera of healthy individuals and cancer patients (29–32, 124). Cancers with elevated circulating levels of soluble form of BTLA include HCC, pancreatic adenocarcinoma, clear cell renal cell carcinoma and prostate cancer (29–32, 124). sBTLA levels was significantly associated with aggressiveness and progression in prostate cancer (32). Association with worst prognosis was observed in HCC patients receiving sorafenib, pancreatic adenocarcinoma, and clear cell RCC (29, 31, 124). Interestingly, these studies had investigated a large panel of co-stimulatory and co-inhibitory immune checkpoint molecules in which only sBTLA level was significantly correlated with cancer prognosis (29, 31, 124). These outcomes indicate a broader regulatory role for BTLA in cancer as compared to PD-1 and CTLA-4 checkpoint axis.

Soluble form is produced as a result of alternative splicing and its transcripts could be detected in B, CD4+ and CD8+ T cells which may constitute the source of sBTLA (23, 24, 260). Two splice variants for BTLA have been reported in mice and humans. One isoform lacking Ig domain and the other one lacking transmembrane domain have been identified (23, 24, 260). Circulating soluble BTLA may possess biological activity and clinical significance (260). But its significance in this regard has not been elucidated in cancer studies (262, 263). However, in a similar manner to sPD-1 experiments, blockade of BTLA-HVEM with sBTLA have shown enhanced anti-cancer immunity in *in vitro* and *in vivo* studies (262). A eukaryotic expression plasmid (psBTLA), which expressed the extracellular domain of murine BTLA with capability to bind HVEM and disrupt BTLA-HVEM interactions, was constructed and its injection resulted in down-regulation of IL-10 and TGF-β and promotion of dendritic cell function *via* increased expression of B7-1 and IL-12. Furthermore, its combination with HSP70 vaccine induced a potent anti-tumor immunity by increasing the expression of Th1 cytokines, IL-2, and IFN-γ and decreasing transcription levels of IL-10, TGF-β, and Foxp3 in the tumor microenvironment (262). Similar results were obtained when a recombinant adeno-associated virus (AAV) vector was used for sBTLA expression in a melanoma pulmonary metastasis model (263). Nonetheless, the research into this checkpoint is at preliminary stages and further exploration would be needed to establish its role as predictive and prognostic marker, as well as, its potential for anti-cancer immunotherapy.

## Conclusions

Immune checkpoint blockade, in particular blocking the CTLA-4 and PD-1 checkpoint pathways, represents a revolutionized form of cancer immunotherapy. Generation of soluble forms of B7:CD28 family coinhibitory checkpoint molecules represents a broader involvement of these pathways in regulation of anti-cancer immunity and further adds to the complexity of pathological interactions exist among these pathways. Soluble forms of these receptors and ligands show their significance in the form of biomarkers for prognosis and prediction of response to therapy but also open new opportunities for anti-cancer immunotherapy. *In vitro* and *in vivo* evidence suggest great potential for soluble forms of PD-1 and B7-1 as therapeutic agents. However, these results have not been validated in clinical studies. Soluble PD-L1 levels ascertains itself as a predictive and prognostic biomarker in cancer progression and remission but also in determination of response to therapy. It becomes imperative to investigate whether reducing sPD-L1 therapeutically could yield in diminution of its inhibitory effects and induction of anti-cancer immune responses. Moreover, their clinical significance may depend on the primary functions of their membrane-bound counterparts and regulatory mechanism and effects of the whole checkpoint pathway. For example, sCTLA-4 elevated levels increase the T cell inhibition and its blockade results in vice versa due to the competitive mechanism of the checkpoint CD28/CTLA-4/B7-1/B7-2 pathway operates. But this is not case for sPD-1 as increasing sPD-1 disrupt the PD-1/PD-L1 pathway and yields in better anti-cancer immunity. The opposite is accurate for their corresponding ligands, increasing sB7-1 results in improved immunity while increasing sPD-L1 is related to worst prognosis. Hence, deep understanding of the regulatory mechanism of primary membrane bound checkpoint pathways can help us understand the mechanism of action and biological effects of soluble checkpoint receptor and ligands. Nonetheless, additional mechanism may also exist as in the case of B7-1. Newer pathways, in particular the BTLA-HVEM, may have broader significance as it appears to regulate all phases of T cell activation. Therefore, in addition to their predictive and prognostic value, understanding the underlying biological mechanism of its production and function of these soluble forms may pave the way for innovative checkpoint-based cancer immunotherapy.

## Author Contributions

All authors listed have made a substantial, direct, and intellectual contribution to the work, and approved it for publication.

## Conflict of Interest

The authors declare that the research was conducted in the absence of any commercial or financial relationships that could be construed as a potential conflict of interest.

## Publisher’s Note

All claims expressed in this article are solely those of the authors and do not necessarily represent those of their affiliated organizations, or those of the publisher, the editors and the reviewers. Any product that may be evaluated in this article, or claim that may be made by its manufacturer, is not guaranteed or endorsed by the publisher.

## Abbreviations

B7, B7 proteins; CD28, cluster of differentiation 28; CTLA-4, cytotoxic T-lymphocyte-associated protein 4; PD-1, programmed cell death protein 1; B7-H3, B7 homolog 3, B7-H4, B7 homolog 4; BTLA, B- and T-lymphocyte attenuator protein; APCs, antigen presenting cells; MHC, major histocompatibility complex; TCR, T cell receptor; ICOS, inducible T cell co-stimulator; B7-H1, B7 homolog 7; PD-L1, programmed cell death 1 ligand 1; B7-DC/PD-L2, Programmed cell death 1 ligand 2; HVEM, herpes virus entry mediator; NK, natural killer cells; IL-2, interleukin 2; IFN- γ, interferon gamma; TACE, trans-arterial chemoembolization; MPM, malignant pleural mesothelioma; HCC, hepatocellular carcinoma; RT, radiation therapy; CRT, chemoradiotherapy; MLRs, mixed lymphocyte reaction; CLL, chronic lymphocytic leukemia; MCL, mantle cell lymphoma; EGR, epidermal growth factor receptor; NF-κB, nuclear factor kappa B; MAPK, mitogen-activated protein kinase; ccRCC, clear cell renal cell carcinoma; AML, acute myeloid leukemia; TM, transmembrane; MDS, myelodysplastic syndrome; CR, complete remission; NSCLC, non-small cell lung carcinoma; NPC, nasopharyngeal carcinoma; DLBCL, diffuse large B- cell lymphoma; HBV, human hepatitis B virus; TKIs, tyrosine kinase inhibitors; PFS, progression free survival; OS, overall survival; IMRT, intensity modulated radiotherapy; HLA-I, human leukocyte antigen class I; PMBCs, peripheral blood mononuclear cells; TNF-α, tumor necrosis factor alpha; CTLs, cytotoxic T lymphocytes; iNOS, inducible nitric oxide synthase; mAb, monoclonal antibody; MMP, matrix metalloproteinase; DCs, dendritic cells; OSCC, oral squamous cell carcinoma; MM, multiple myeloma; NKTTL, nasal NK/T cell lymphoma; EOC, epithelial ovarian cancer; DFS, disease free survival; SBRT, stereotactic body radiotherapy; BTC, biliary tract cancer; MDDCs, monocytes-derived dendritic cells; AFP, alpha feto protein.
